# Pediatric residents' experiences of a clinical rotation in Adolescent Medicine

**DOI:** 10.1186/1472-6920-10-88

**Published:** 2010-12-01

**Authors:** Fadia AlBuhairan, Karen Leslie, Eudice Goldberg

**Affiliations:** 1 Department of Pediatrics, King Abdulaziz Medical City and King Saud bin Abdulaziz University for Health Sciences, Riyadh, Saudi Arabia; 2 Division of Adolescent Medicine, Department of Pediatrics, The Hospital for Sick Children and University of Toronto, Toronto, Canada

## Abstract

**Background:**

Although Adolescent Medicine is a pediatric subspecialty, it addresses many issues that differ from other aspects of pediatrics clinical training. The aim of this study was to explore the general experiences of pediatric residents during their rotations in Adolescent Medicine.

**Methods:**

Qualitative methods were applied. Semi-structured individual interviews were conducted with pediatric residents who had completed a rotation in Adolescent Medicine. Emergent themes were identified.

**Results:**

Three key themes emerged: gaining exposure, taking on a professional role, and achieving self-awareness. Subcategories were also identified. There was particular emphasis on the multidisciplinary team and the biopsychosocial approach to adolescent health care.

**Conclusions:**

The experiences in Adolescent Medicine reflected residents' learning, notably gains in the "non-expert" as well as "medical expert" physician competencies. Future studies should explore how the interprofessional nature of an Adolescent Medicine team and the patient populations themselves contribute to this learning.

## Background

Adolescent Medicine is a pediatric subspecialty that addresses clinical issues that are often unique and different from other aspects of training in pediatrics. Pediatric postgraduate training programs in North America are required to provide at least a one-month block in Adolescent Medicine [1,2] for trainees. During this training, residents may be exposed to ethically challenging or controversial issues and to patients who are stereotypically labeled as being 'difficult'. Unlike with younger children, trainees most often communicate directly with the adolescent patient for both assessment and management issues.

Some studies in the literature have examined pediatric residents' and other trainees' clinical skills and/or knowledge of adolescent health care [3-8]. Others have assessed pediatric residency training programs for the adolescent medicine training provided to their residents [9], and some have even looked at physicians' personal adolescent experiences or values and their subsequent effects on the delivery of health care to adolescent patients [10,11]. However, no studies to date have explored the experiences and perceptions of residents themselves during their postgraduate training in Adolescent Medicine and how such clinical experiences and exposure to different patient populations and patient-provider interactions may differ from the rest of their training in pediatrics.

The aim of this study was to explore the general experiences and possible challenges faced by pediatric residents during their rotation in Adolescent Medicine. Such information may be used to a) better address the needs of residents during this rotation; b) create an environment that supports learning; and c) develop curricula that foster the development of clinical competence in the provision of care to adolescents. By addressing these issues, one hopes that the ultimate goal of providing quality patient care and improved patient outcomes is achieved.

## Methods

Because of the nature of the question and the lack of literature on the topic, an exploratory, qualitative design, as described by Strauss and Corbin [12], was adopted for this study.

### Setting

The study was conducted between August 2006 and July 2007 at an academic tertiary care pediatric hospital in Canada, where the Division of Adolescent Medicine provides outpatient and inpatient services for a range of adolescent issues. Approval for the project was obtained from the institution's Research Ethics Board.

### Participants

As part of their core training, pediatric residents have a four-week rotation in Adolescent Medicine during their first year of residency (PGY1). Using *convenience sampling *[13], all PGY1 pediatric residents who completed their rotation were asked to participate in the study. All other trainees were excluded. Eligible participants were contacted by a research assistant external to the Division. Each resident was contacted at the end of his/her rotation, after his/her end-of-rotation evaluation had been completed and submitted. Written consent was obtained from all interested participants. Recruitment continued until *theoretical saturation *[12,13] was achieved.

### Data collection

Data was collected using open-ended, semi-structured, individual interviews, which were approximately 30-60 minutes in duration. The interview questions were prepared and tested beforehand and modified accordingly. Interviews were audio-recorded and conducted in a private setting by the same research assistant who recruited the participants. The interviewer also took field notes and collected demographic information from each participant.

Audiotapes were transcribed verbatim with the exclusion of all identifying data. Two of the interviews were not audio-recorded because of technical problems; analyses of those two interviews were based on the information gathered from the field notes. Because only 1-2 residents rotated through Adolescent Medicine each month, transcripts were randomly assigned non-consecutive numbers for identification to further ensure participants' anonymity.

### Data analysis

Data analysis occurred alongside data collection, a method referred to as the *constant comparative method *[13]. Three transcripts were analyzed at a time to ensure participants' confidentiality and anonymity.

Transcripts were independently analyzed for emergent themes by all three investigators, and a manual coding structure was developed through group negotiation [12,13]. Analysis occurred in a hierarchical manner with the identification of codes, concepts, and themes. When new themes failed to emerge, *theoretical saturation *[12] was considered to have been achieved, and no additional participants were recruited.

Findings from the initial analysis were shared with a representative number of the study participants on an individual basis. These *member checks *[13] provided additional validation of the data.

## Results

Of all eligible trainees approached to participate, one declined and two others agreed but did not confirm interview appointments, even after several attempts were made to schedule them. Theoretical saturation was achieved by conducting interviews with 13 participants, 7 males and 6 females. Their mean age was 28.8 years, with a range of 25-42 years. Five participants received their medical education in countries other than the USA and Canada.

Three key themes emerged in the data (Table 1): 1) **gaining exposure**, 2) taking on a **professional role**, and 3) achieving **self-awareness**. These three themes were further subcategorized and are reviewed below.

**Table 1:** Emergent themes

*Themes*	*Subthemes*
*Gaining exposure*	Knowledge
	Insight
	Comprehensiveness
*Professional role*	Relationship with patient
	Engagement
	Advocacy
	Feelings
	Role within health care team
	Support
	Communication
*Self-awareness*	Personal values
	Past personal experiences
	Attitudinal shift

### Gaining Exposure

#### Knowledge

Participants spoke of their exposure to adolescents and described gaining knowledge about various adolescent issues, including the development and behavior of adolescents. Residents expressed enhanced insight into the complexity and reality of adolescents' lives and decisions: "*The lived experience of having actually worked with the conditions that I've read about and putting that into practice certainly made my feelings about the conditions and the adolescent's suffering from them more real" *(Participant 3).

#### Insight

The insight gained through their experiences with adolescents with chronic illness was described in the following quote:

*Like, they (adolescents with chronic illness) have an active home life and lifestyle, but at the same time the medical aspects impact on those other areas in some way or another. It's hard to describe in words, but a sense I guess of wonderment too that these guys have gone through so much hardship through their childhood, were learning now how to engage with other peers who probably had not had that kind of experience, and try to be as normal as possible, where they may not appear normal to their peers... *(Participant 2).

#### Comprehensiveness

The biopsychosocial approach to adolescent health care was regularly brought up by participants. This holistic and comprehensive approach was typical of the care provided to adolescents during this rotation, as opposed to strictly focusing on the main concern/problem as residents had experienced in other areas of pediatric medicine. As one participant said: *"...an advantage of the rotation is that we don't really have much exposure to adolescents in other areas of the hospital, and when we do, it's very focused on their medical issue and not looking at like everything else..." *(Participant 1).

Another resident referred to Adolescent Medicine as a *"crossroad of medical issues and psychosocial issues" *(Participant 2), reflecting the comprehensive nature of the care provided.

### Taking on a Professional Role

The second theme that emerged in this study was the professional role that participants acquired. Two subcategories emerged within this theme: the professional relationship with the adolescent patient and the role of the resident within the health care team.

#### Relationship with patient

Residents felt that as a result of their experiences in their rotations in Adolescent Medicine, they were better able to establish rapport and engage adolescent patients. Residents discussed directly communicating with the adolescents, rather than through their parents, and approaches for communicating with adolescents were individually tailored to meet the needs, personalities, and developmental stages of the adolescent patients. One of the study participants commented:

*I feel more comfortable working with them. ...[a]nd now my out-patient (who was previously) in the unit is saying 'can you be my doctor? Can you be my doctor?' Because you know, ... getting along with them, talking with them, know how do they feel, how do they think at this age*... (Participant 5).

Residents' desire to help this patient population was evident: *"...and you felt that you should be there all the time helping, not just for medical issues and besides emotional. Then just helping to figure out what's going on and find out what is the best way for them" *(Participant 5). Participants also identified an advocacy role with their adolescent patients: "... *[a]nd they (adolescents) have rights, especially rights to decide for themselves" *(Participant 5).

The participants described a variety of feelings toward their adolescent patients. A participant shared how he felt after an interaction with a teen mom:

*Frustration. A little bit of shock at some of the presentations, and sadness. But also significant and profound moments of connection and happiness that headway was being made or that understanding seemed to be created and a therapeutic bond developed *(Participant 3).

Other residents also reported feeling frustrated at times. These feelings of frustration were usually related to patients not adhering to treatment recommendations and appointment scheduling, as adolescent patients were often either late to or did not attend their appointments. Frustration was also expressed toward patients with eating disorders; this seemed to be related to participants' lack of understanding of the underlying pathology: *"[a]nd I was frustrated because she was choosing, I felt, to take on a sick role. And it was her choice. And I didn't know. I thought it was all behavior and not organic in origin" *(Participant 13).

Participants also shared the positive feelings and satisfaction they experienced in working with adolescents patients: "*I looked forward to coming into work to work with them (the patients)*" (Participant 9).

#### Role within health care team

The role of the resident within the health care team was the second subcategory that emerged within this theme. Involvement with an interprofessional team not only aided in the comprehensive appreciation of the issues from various viewpoints, but also provided a supportive environment for the trainees: *"I think the team's been really good in a confidential way, just in discussing (issues) among health care professionals in the team of sharing emotions and kind of venting that way, which I think is a professional way to do things" *(Participant 11).

Participants regularly communicated with other members of the team, whether nurses, social workers, dietitians, or other physicians. Participants viewed the rest of the team as providing a supportive, safe and healthy learning environment and recommended that future trainees discuss challenging situations with members of the health care team. One of the residents made the following statement: *"...there is a profound amount of wisdom to be learned from all the people you're working with" *(Participant 3).

### Self-Awareness

The final theme that emerged was self-awareness. All of the participants were reflective of their experiences in Adolescent Medicine and were conscious of their personal values and beliefs. They expressed an awareness of their own experiences as adolescents and were aware of shifts in their attitudes toward adolescents and/or Adolescent Medicine.

#### Personal values

All participants identified some of their personal values and biases; for some, these were consistent with those of their adolescent patients, while for others, there was tension between their own personal values and those of their patients. For the latter group, there were some residents who were aware of this conflict and believed that it was necessary to be nonjudgmental and offer adolescents all possible options, even if these options conflicted with their own personal values and beliefs. These residents also felt that it was important to try not to influence adolescents with their own personal beliefs/values. For others who had conflicting values, this conflict posed a significant challenge, and a resident's main coping mechanism was to avoid or not take part in the situation: *"I think I avoided situations that would have been the most difficult, or situations that I would not have been able to handle. So no, I think I was just consciously aware of situations and didn't want to be part of (them)" *(Participant 11).

Other forms of coping strategies identified by residents included discussing situations with other members of the health care team, with a member of their own family, or utilizing faith-based support. The specific clinical scenarios that were avoided by a few of the residents, because of conflicting personal values and beliefs, were situations involving discussions about contraception and/or counseling a pregnant teenager.

#### Past personal experiences

Participants were also reflective of their own adolescent experiences: *"I don't think that anyone could say that their adolescence didn't influence it (the experience in Adolescent Medicine) in some way. Like everyone's experience affects every subsequent experience" *(Participant 1).

Many of the participants compared their adolescent lives and experiences to those of their patients, with some being consciously aware of their internal biases: *"... (I was) a bit judgmental on how teens act today compared to when I was a teenager. Like the things that they dare said, or like the drugs they took, the amount of people they slept with..." *(Participant 13).

Others spoke about trying to maintain a more neutral standpoint: *"(I) kept an open mind and didn't assume anything about any of the patients that I met" *(Participant 2).

#### Attitudinal shift

Participants were also aware of their attitudinal shifts. Several had preconceived ideas about adolescents and/or Adolescent Medicine, which shifted during the rotation. These attitudinal shifts were generally positive: *"... I was kind of a bit scared...but then when I got to know them (street involved youth) better, they're actually more friendly than I thought they are. They're just teenagers and they're very nice essentially" *(Participant 12).

This general sense of self-awareness prompted several participants to recommend that future trainees begin the rotation with an open mind and a conscious awareness of their own beliefs. As one participant advised, *"keep as broad and as open a mind as possible..." *(Participant 3), and another resident stated, *"...you really have to keep an open mind and see how to best work with these kids, learn from these kids, and how you can alter your own approach and your own practice in working with these adolescents to the best of your ability for the best possible outcome" *(Participant 7).

## Discussion

This study contributes to our understanding of the experiences of pediatric residents during their postgraduate training in Adolescent Medicine in several ways.

It is not surprising that residents gained knowledge through this training, as it is expected that people will learn as the result of any given experience, and other researchers have similarly reported the improvement in knowledge and clinical skills in adolescent health after participating in such a rotation [8]_. _The experience during this rotation, however, went beyond a simple gain in knowledge; residents gained insight and a comprehensive understanding of adolescents' lives and issues. They acted on the wealth of information that they had acquired and the skills that they had learned by engaging with and advocating for their adolescent patients. They communicated and collaborated with other members of the interprofessional health care team, and finally, they reflected upon their whole experience.

In other words, this rotation not only assisted residents in becoming the "medical expert" but also assisted residents in developing some of the "non-expert" physician competencies that postgraduate medical education programs aim to achieve. CanMEDS physician competencies [14] exemplify such a framework, and like other similar frameworks, include competencies such as communicator, collaborator, health advocate, and professional. These competencies have been developed with the ultimate goal of improving patient care. They were not specifically assessed or evaluated in this study, but interestingly, the residents' experiences during their Adolescent Medicine rotation reflected these areas that are essential components of postgraduate and other medical education training programs.

The biopsychosocial and comprehensive approach to adolescent patients was repeatedly mentioned by the residents. They compared this experience to other areas of training in pediatrics in which such an approach was not consistently modeled. Trainees were generally familiar with a problem-focused approach, where a patient's chief complaint was addressed and other aspects of a patient's life not routinely explored. With the complexity of adolescents' issues, the residents recognized that focusing solely on the chief complaint would be inadequate and/or misleading, as one might not get the opportunity to really 'get to know' his/her patient in so doing.

Patient care, in general, is known to be complex and requires that multidisciplinary professionals work together in an effective manner to deliver quality care [15]. Interprofessional education (IPE) has been suggested as a way of improving interprofessional collaboration and patient care, yet the results of health outcomes are mixed [15]. The training in Adolescent Medicine was not of an IPE nature, meaning that students were not of different professional backgrounds, yet the learning occurred in an interprofessional team environment. Residents' roles within the interprofessional health care team were established. The value of communicating and collaborating with the team in managing challenging situations, seeking resources, or discussing particular clinical experiences was reported. The interprofessional nature of the team allowed for a variety of perspectives on issues and complemented the biopsychosocial approach to patient care. Trainees learned from the other health professionals and their respective roles in the care of their patients. The team was also considered a source of support for trainees when they encountered a clinically or ethically challenging situation. The interprofessional team environment could serve as a model for other postgraduate medical training programs and may be used to assess the role of the interprofessional team environment in the learning of "non-expert" physician competencies.

Engaging with their adolescent patients promoted feelings of empathy, and the trainees' roles as patient advocates became increasingly evident to them. Participants who previously acknowledged being indifferent toward adolescents and their behaviors expressed a shift in these attitudes and described an enhanced awareness and understanding of adolescent behavior. The mixed feelings that emerged reflect the countertransference that is known to be a part of the doctor-patient relationship [16,17]_. _Not only was there a shift in attitude toward adolescents, but there was also a shift in attitudes toward Adolescent Medicine, with a few participants reporting that they were positively influenced by their rotation and were now considering Adolescent Medicine as a future career path. One might wonder if trainees had not previously considered Adolescent Medicine as a future specialty because of a lack of exposure to this area or because of negative preconceived ideas about working with adolescents.

The exposure of participants to potentially controversial issues during their rotation, as well as the nature of the interviews that were conducted for this study, might have resulted in the heightened self-awareness that all participants described. Most of the participants were in their mid to late 20s; the age difference between them and the adolescent patient population was not considerable, which could have contributed to participants reflecting on their own adolescent experiences.

The fact that some participants recommended that future trainees commence their rotation in Adolescent Medicine with an open mind and a self-awareness of their own values and biases is consistent with published reports about clarification of values and how this may assist with decision making [11,18]. Cultural stereotypes about teenagers, as well as personal and ideological beliefs and values, are known to shape a clinician's approach to adolescent patients [11,19]. Furthermore, the personal backgrounds, values, and own adolescent experiences of residents have been associated with their attitudes about adolescents and their approach to adolescent health care [10,11]. Being conscious of one's opinion can assist a trainee in his/her approach to patient management as well as facilitate discussion with members of the health care team. Awareness of physicians' feelings and responses toward their patients is known to impact the quality and character of the doctor-patient relationship [10,18].

**Figure 1:**
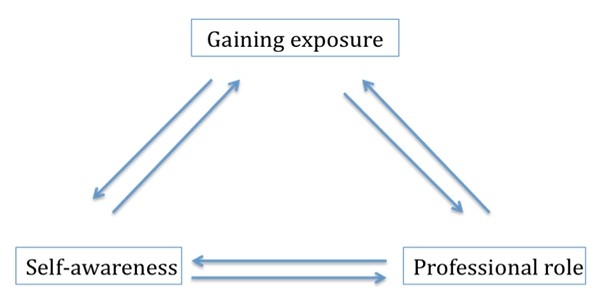
**Potential model of the learning process**. The three key themes identified, *gaining exposure*, taking on a *professional role*, and *self-awareness*, may represent elements of a learning process and may be related to one another in a bi-directional, 'triangular' manner.

In essence, pediatric residents' experiences during their clinical rotation in Adolescent Medicine may have reflected a *learning process*. Residents gained knowledge and insight through clinical exposure, took on a professional role as a member of a health care team, and reflected through self-awareness. One may wonder if these elements were related and if so, if they occurred in series, in a unidirectional or bidirectional manner. Figure 1 illustrates a potential model of such a learning process. Though unclear if and how each of these elements led to the next, residents, through their experiences, built their "non-expert" physician competencies in addition to their "medical expert" physician competencies in an interprofessional team environment.

This particular gain in the "non-expert" physician competencies during the rotation may support the need for pediatric residents and/or other specialty residents to take their Adolescent Medicine rotation during PGY1, as this exposure and learning may enrich subsequent clinical experiences with adolescent patients seen during other rotations where the biopsychosocial approach to adolescent health care may not be modeled.

This study's findings reflect the experience at one particular institute with diverse patient and trainee populations. Therefore, these findings may not be generalizable to all Adolescent Medicine training programs. Other centers, nationally or internationally, may have a different trainee and/or adolescent patient population that could influence trainee experiences. The methodology utilized, however, could be used for further studies examining and comparing resident and other trainee experiences in clinical settings.

## Conclusions

Pediatric residents' experiences of their clinical rotations in Adolescent Medicine reflected their learning, notably gains in the "non-expert' as well as "medical expert" physician competencies. Future studies should explore the proposed potential model of the learning process, whether this learning is similar in other Adolescent Medicine training programs and in other specialty/subspecialty postgraduate medical training programs, and whether the interprofessional nature of an Adolescent Medicine team and the patient populations themselves contribute to this learning. Further studies could also explore the effect of values clarification prior to engaging in an Adolescent Medicine rotation, as a way of 'priming' the learning experience and might inform the development of orientation materials for future trainees.

## Competing interests

The authors declare that they have no competing interests.

## Authors' contributions

FA conceived of the study, participated in its design and analysis, drafted the manuscript, and obtained the funding. KL and EG participated in the design and analysis of the study, as well as in the critical review of the manuscript. All authors read and approved the final manuscript.

## Pre-publication history

The pre-publication history for this paper can be accessed here:

http://www.biomedcentral.com/1472-6920/10/88/prepub
